# Distinct Kinematic and Neuromuscular Activation Strategies During Quiet Stance and in Response to Postural Perturbations in Healthy Individuals Fitted With and Without a Lower-Limb Exoskeleton

**DOI:** 10.3389/fnhum.2022.942551

**Published:** 2022-07-15

**Authors:** Charles S. Layne, Christopher A. Malaya, Akshay S. Ravindran, Isaac John, Gerard E. Francisco, Jose Luis Contreras-Vidal

**Affiliations:** ^1^University of Houston, Houston, TX, United States; ^2^Center for Neuromotor and Biomechanics Research, College of Liberal Arts and Social Sciences, University of Houston, Houston, TX, United States; ^3^Noninvasive Brain-Machine Interface System Laboratory, Department of Electrical and Computer Engineering, University of Houston, Houston, TX, United States; ^4^TIRR Memorial Hermann and Department of PMR, University of Texas Health Sciences Center, Houston, TX, United States

**Keywords:** exoskeleton, posture, EMG, kinematics, perturbations

## Abstract

Many individuals with disabling conditions have difficulty with gait and balance control that may result in a fall. Exoskeletons are becoming an increasingly popular technology to aid in walking. Despite being a significant aid in increasing mobility, little attention has been paid to exoskeleton features to mitigate falls. To develop improved exoskeleton stability, quantitative information regarding how a user reacts to postural challenges while wearing the exoskeleton is needed. Assessing the unique responses of individuals to postural perturbations while wearing an exoskeleton provides critical information necessary to effectively accommodate a variety of individual response patterns. This report provides kinematic and neuromuscular data obtained from seven healthy, college-aged individuals during posterior support surface translations with and without wearing a lower limb exoskeleton. A 2-min, static baseline standing trial was also obtained. Outcome measures included a variety of 0 dimensional (OD) measures such as center of pressure (COP) RMS, peak amplitude, velocities, pathlength, and electromyographic (EMG) RMS, and peak amplitudes. These measures were obtained during epochs associated with the response to the perturbations: baseline, response, and recovery. T-tests were used to explore potential statistical differences between the exoskeleton and no exoskeleton conditions. Time series waveforms (1D) of the COP and EMG data were also analyzed. Statistical parametric mapping (SPM) was used to evaluate the 1D COP and EMG waveforms obtained during the epochs with and without wearing the exoskeleton. The results indicated that during quiet stance, COP velocity was increased while wearing the exoskeleton, but the magnitude of sway was unchanged. The OD COP measures revealed that wearing the exoskeleton significantly reduced the sway magnitude and velocity in response to the perturbations. There were no systematic effects of wearing the exoskeleton on EMG. SPM analysis revealed that there was a range of individual responses; both behaviorally (COP) and among neuromuscular activation patterns (EMG). Using both the OD and 1D measures provided a more comprehensive representation of how wearing the exoskeleton impacts the responses to posterior perturbations. This study supports a growing body of evidence that exoskeletons must be personalized to meet the specific capabilities and needs of each individual end-user.

## Introduction

Exoskeletons are increasingly being used to promote effective gait across a variety of populations. However, the postural stability of individuals using lower limb exoskeletons for gait assistance may be compromised and they will therefore be more susceptible to falling (He et al., 2017). In order to maintain standing balance, there should be a harmonious relationship between the exoskeleton and the human user, making it necessary to integrate the knowledge of human balance control in exoskeleton development (Emmens et al., 2018). To date, lower limb exoskeletons have few, if any, features designed to mitigate falls (He et al., 2017; Monaco et al., 2017; Bayón et al., 2022). Mummolo et al. (2018) emphasized the need for exoskeletons to include stabilization features to prevent user falls. Moreover, they stress that in order to develop stable robotic exoskeletons, quantitative information regarding the stability of the exoskeleton in concert with the user is necessary from the initial design until completed production. Thus, it is important in future designs to develop “user-in-the-loop” features that support improved postural control, including the use of brain-machine interfaces (BMIs; Contreras-Vidal et al., 2018; He et al., 2018; Kilicarslan and Contreras-Vidal, 2021).

While working to develop exoskeletons with improved stability features, it is also important to remember that users will have varying abilities and unique responses to postural challenges due to age, neurological state, brain or body injury, physical disabilities, changing environments, and other factors. As reported by Bortole et al. (2015), individuals interacting with an exoskeleton displayed idiomatic response patterns. Echoing this point, Fan and Yin (2013) found that the coordination between force and position between individuals and exoskeletons was variable across individuals. This suggests that effective exoskeletons need to be personalized to meet the specific and possibly evolving capabilities and needs of each individual end-user.

Support surface perturbations have long been used to characterize the postural response characteristics of humans to the loss of balance (Nashner, 1977; Gera et al., 2016; Goel et al., 2022). Perturbation-based research provides controlled environments in which an investigator can control multiple characteristics of the perturbation, such as direction, magnitude, and timing, as well as the number of trials. Moreover, it allows for multiple sensor technologies to be simultaneously used to collect kinematic, force, and neurophysiological data, such as electromyography (EMG) and electroencephalography (EEG); this provides an efficient paradigm with which to study the neural basis of postural control. Studying the responses of healthy individuals wearing lower limb exoskeletons during support surface perturbations can provide important insights and normative data into how humans adapt to postural control while wearing an exoskeleton (Schiffman et al., 2008; Fasola et al., 2019; Ringhof et al., 2019).

Oftentimes, scientists explore potential differences in time-based waveforms by using discrete 0 dimensional (0D) measures such as peaks, minimums, maximums, or the mean values of those measures. However, these discrete measures can fail to identify important features of time series, such as pattern shape, and are limited in their capability to detect differences between conditions or participant populations. Statistical parametric mapping (SPM) is an increasingly used technique to evaluate potential differences between time varying (1D) waveforms such as kinematic or muscle activation data. SPM enables the comparisons of entire waveforms by accounting for the dependency of adjacent samples in the calculation of appropriate alpha levels (Pataky et al., 2013). In this study, in addition to using several discrete measures, SPM was used to explore potential differences in COP and EMG waveforms with and without wearing an exoskeleton.

The long-term goal of this project is to use an individual’s brain waves, acquired *via* scalp electroencephalography (EEG), to identify an impending fall and use that information to activate an exoskeleton to produce the torques necessary to prevent said fall. However, prior to realizing this aim, a greater understanding of how wearing an exoskeleton impacts postural control is necessary, particularly given that behavioral responses are individualized depending upon a person’s unique abilities. Fully characterizing responses to postural perturbations with and without wearing an exoskeleton will provide engineers with the information necessary to develop the next generation of exoskeletons with improved postural control features. In this article, we report center of pressure (COP) and surface electromyography (EMG) results obtained in response to a series of posterior standing perturbations with and without wearing a lower-limb exoskeleton. Recent companion reports detail the progress being made in using single perturbation trial EEG to predict impending falls (Ravindran et al., 2020, 2022).

## Materials and Methods

### Participants

Seven healthy adults (five males) aged 24.8 ± 2.8 years, with a mean weight of 72.9 ± 14.3 kg and a mean height of 66.9 ± 2.8 cm, participated in this study. Inclusion criteria included being free of any physical, neuromuscular, or vestibular-related issues that may impact postural control as well as being between the ages of 18–35 years old. The experimental protocol was approved by the Institutional Review Board (IRB) at the University of Houston, in accordance with the Declaration of Helsinki. Each participant provided written informed consent.

#### Instrumentation

After a thorough cleaning of the skin, surface electromyographic (EMG) electrodes (Delsys, Natick, MA, USA) were affixed bilaterally over the lateral gastrocnemius (LG), medial gastrocnemius (MG), soleus (SO), and tibialis anterior (TA). A wireless DelsysTrigno system was used to collect EMG data. The participants were also instrumented for 64 channel EEG data collection. A complete description of the EEG instrumentation and data collection procedures can be found in Ravindran et al. (2020).

An H2 exoskeleton (Technaid S.L., Madrid, Spain) in passive mode with the joints uncoupled was used during testing. The H2 includes bilateral hinged hip, knee, and ankle joints with articulated footplates as well as waist support. Uncoupling of the exoskeleton joints means that the motors present at each joint (hip, knee, and ankle) were not activated during the experiment, and thus did not exert any force on the system itself, nor provide any resistance, or assistance to the participant. The entire system weighs 11 kg. For a more complete description of the H2, see Bortole et al. (2015). After instrumentation, participants were fitted in the exoskeleton by aligning the robot’s articulated joints with the hip, knee, and ankle joints of the participants, and provided 5 min with which to become accustomed to the device. During this time, the participants slowly walked around an open laboratory space. Participants then stepped onto a Neurocom Balance Master (NeuroCom, Clackamas, OR, USA) and, once positioned in accordance with Neurocom’s recommendations, each individual’s feet were outlined on the plate with the use of adhesive tape. This ensured that each participant could be placed in the same position for all testing conditions.

#### Collection Procedures

**Figure 1:**
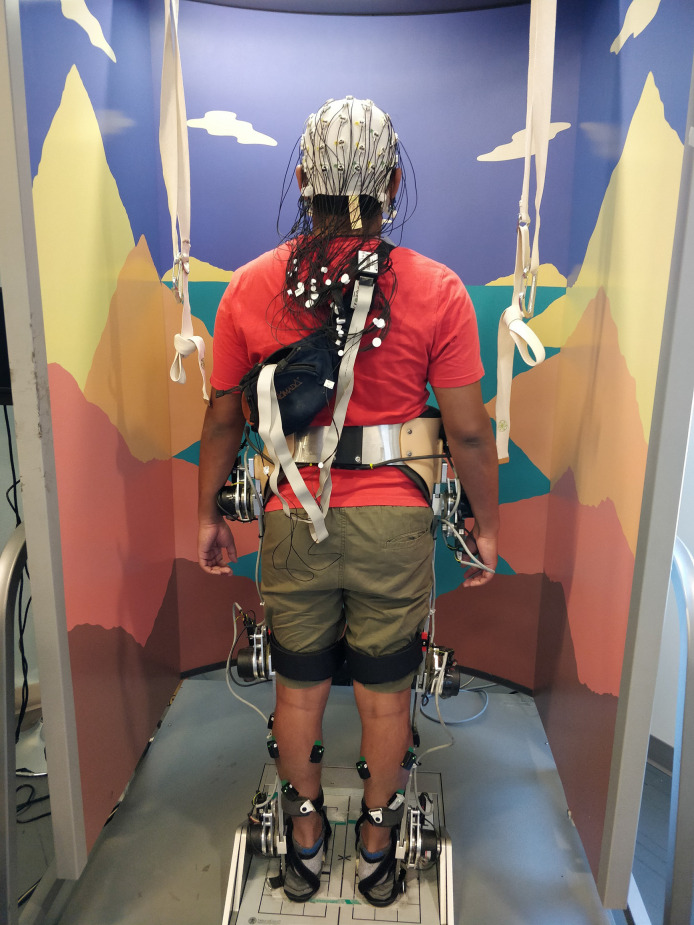
A fully instrumented participant prepared for data collection with and without wearing the H2 exoskeleton while standing on the Neurocom Balance Master. Note the foot pads on H2. During data collection, the participant was secured in a harness to prevent falling.

In order to determine if wearing the H2 exoskeleton modified bipedal static balance, data collection began with a 2-min static balance test, with and without wearing the exoskeleton. Participants were then tested using posterior support surface perturbations with displacements of 6.35 cm, 400 ms duration, and a velocity of 15.875 cm/s. Each participant experienced 32 (two blocks of 16) posterior postural perturbations (Figure 1). Prior to the perturbation it was ascertained that the participants were stable and the perturbation onset delivered randomly within a 2 s window thereby preventing anticipatory behavior. Each individual perturbation window (from onset to full plate recovery) lasted a total of 5 s. Participants were provided a seated break to prevent possible fatigue after the first block of trials. Testing was conducted both with and without the H2, with four participants testing first with the H2, and three testing without the H2, before moving to the opposite condition. Force plate data were sampled at 100 Hz and EMG data was collected at 1,111.1 kHz. The collection technologies were synchronized using a signal from the Balance Master at the beginning of each trial.

#### Data Processing

Kinetic data collected from the Balance Master were used to compute each participant’s COP. Sagittal plane COP was used to characterize the kinematic response to the perturbation. EMG data were bandpass filtered using a 20–450 Hz, 4th order Butterworth filter. The filtered data were then rectified and passed through a 40 Hz low pass filter before being down sampled to 100 Hz, matching the COP data. Both the COP and EMG data for each trial were temporally synchronized to the perturbation onset and an analysis window composed of 200 ms prior to and 750 ms after the perturbation onset was identified. The beginning of the analysis window was selected to provide a stable baseline measure prior to the perturbation, and the cut-off time was selected because the COP of all participants had stabilized by 750 ms after the perturbation.

For each participant, the mean waveforms of the COP and EMG for each muscle were computed after removing the first trial of each perturbation block. This was to prevent including the startle response that participants displayed in response to the first perturbation. Therefore, 30 total trials were used to develop the mean waveforms for both exoskeleton conditions (with and without H2). COP waveforms were amplitude normalized such that the first point of each waveform was zero. EMG waveforms for each muscle, for each participant, were amplitude normalized using the mean value of the EMG collected across the two perturbation conditions. Due to the symmetrical responses of the leg muscles to the perturbations, only the EMG from the left leg was analyzed. COP pathlengths were also computed for each perturbation trial (18), as well as COP velocity and position for each exoskeleton condition.

#### Data Analysis

For the 2-min baseline trials, the RMS of the COP and COP velocity over the entire waveforms were obtained for each participant (Prieto et al., 1996; Fasola et al., 2019). For the perturbation trials, a data analysis window of 950 ms, comprised of 200 ms prior to the perturbation onset to 750 ms after the onset, was established. We analyzed the 0D variables by obtaining the peak COP, peak COP velocity, and maximum pathlength within the data analysis window i.e., the final pathlength value in the analysis window for each participant and experimental condition. We also obtained the peak EMG normalized amplitude within the analysis window for each participant, muscle, and condition. Individual participant means and then grand means and standard deviations (SD) were calculated. The Kolmogorov-Smirnov test was used to ascertain that the data were normally distributed. To determine if there were systematic effects of wearing the H2, these 0D variables were tested for potential significant differences using paired t-tests, using an alpha level of 0.05. We also computed confidence intervals (CI) and effect sizes for each set of comparisons.

In preparation for using SPM, we divided the analysis window into three epochs. These epochs reflected significant behavioral responses associated with the perturbations. These consisted of a baseline (200 ms prior to perturbation onset), response (0–350 ms after onset which represents the peak COP value), and recovery (351–750 ms after onset). Using SPM, potential differences in COP, pathlength, and EMG for each muscle, between the two H2 conditions, for each participant, were evaluated. For each participant, the results of the SPM analyses are presented as a percentage of samples within each epoch that are significantly different between the H2 and no H2 conditions.

## Results

In this report, we present the results of the 0D variables in tabular form that includes mean values for each participant and variable, SDs, CI’s, T and P values as well as effect size. SPM testing is an effective technique to assess potentially different strategies by individuals in response to the perturbations and thereby affords additional insights into response strategies beyond what can be deduced from 0D variables. As Bates (1996) stated, single subject assessments are appropriate when “variations in movement are the result of different solutions (strategies) to the same task by individual subjects” (p.633). In concert with the concept that new generations of robotic exoskeletons will require design features that allow for personalization to meet the unique needs of individuals, we report the outcome of SPM procedures, for each of our participants to the postural perturbations.

### 0 Dimension Results

#### Static Balance

Table 1 provides the results of the RMS data and COP velocity calculated across the entire COP waveform of the 2-min static balance test. There was no statistical difference between the two COP means indicating that the magnitude of sway was not impacted by wearing the H2. There was not a significant difference between the COP velocity while wearing the H2 vs. not wearing the H2. However, there was a clear trend (*p* = 0.067) toward increased velocity in the H2 condition. Six of the seven participants demonstrated increased COP velocity in the H2 condition.

**Table 1:** COP and COP Velocity RMS values during 2-min quiet stance with and without wearing the H2 exoskeleton.

**Participant**	**No H2 COP**	**H2 COP**	**No H2 COP Velocity**	**H2 COP Velocity**
1	0.699	0.687	0.0125	0.0427
2	0.672	0.270	0.0170	0.0223
3	0.831	0.805	0.0156	0.0136
4	0.495	0.643	0.0117	0.0165
5	0.345	0.567	0.0123	0.0407
6	1.220	0.681	0.0127	0.0160
7	0.291	0.504	0.0116	0.0175
Mean ± 1 SD	0.651± 0.318	0.594 ± 0.171	0.0133 ± 0.0021	0.0242 ± 0.0123
95% Confidence Interval	0.415–0.886	0.467–0.721	0.0118–0.0149	0.0151–0.0333
T score	−0.498	2.226		
P value	0.636		0.067	
Effect Size	0.222		−1.205	

#### Perturbation Responses

**Figure 2:**
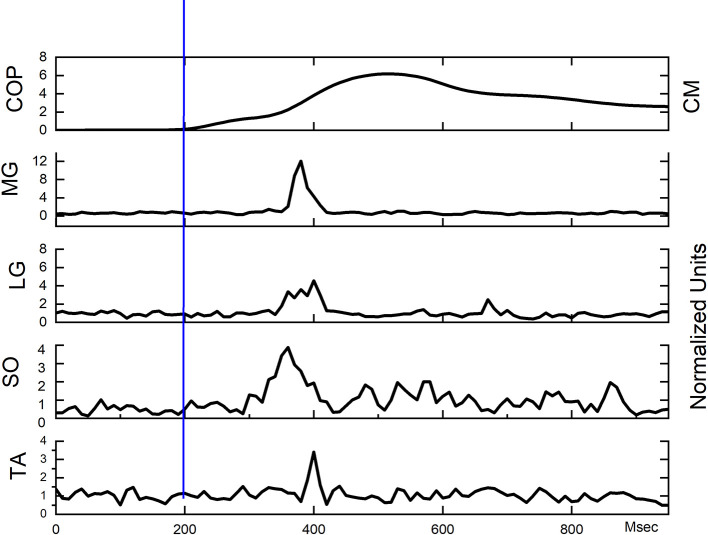
Exemplar COP and EMG waveforms from a single participant. Perturbation onset occurred at 200 on the absciss (blue vertical line).

Figure 2 provides a representative example of the COP and the associated EMG activation patterns in response to the posterior perturbation.

Table 2 displays the mean peak COP, COP velocity, and pathlength for each exoskeleton condition and their associated statistics. All three comparisons between the two exoskeleton conditions were significantly different with all displaying large effect sizes.

**Table 2:** Peak COP, Peak COP Velocity, and maximum Pathlength in response to backward translations with and without wearing the H2 exoskeleton.

**Participant**	**No H2 Peak COP (cm)**	**H2 Peak COP (cm)**	**No H2 Peak COP Velocity (cm/s)**	**H2 COP Velocity (cm/s)**	**No H2 Pathlength**	**H2 Pathlength**
1	7.149	5.848	0.645	0.351	15.56	10.35
2	7.517	7.239	0.512	0.493	16.93	14.74
3	7.547	6.734	0.464	0.406	16.09	12.95
4	8.475	8.926	0.728	0.671	19.16	21.50
5	8.161	6.829	0.556	0.385	17.10	12.68
6	7.125	5.791	0.483	0.411	13.77	10.47
7	7.431	6.733	0.458	0.417	13.67	11.70
Mean ± 1 SD	7.629 ± 0.507	6.871 ± 1.050	0.560 ± 0.102	0.448 ± 0.108	16.04 ± 1.94	13.48 ± 3.85
95% Confidence Interval	7.254–8.005	6.094–7.649	0.474–0.625	0.368–0.527	14.60–17.48	10.63–16.33
T score	−3.014		−2.768		−2.771	
P value	0.023		0.033		0.032	
Effect Size	0.92		0.971		0.839	

Table 3 displays the EMG data and reveals that only the peak MG displayed a significant difference between with or without wearing the H2.

**Table 3:** Peak EMG values for each muscle in response to backward translations with and without wearing the H2 exoskeleton.

**Participant**	No H2 Peak TA*	**H2 Peak TA**	**No H2 Peak MG**	**H2 Peak MG**	**No H2 Peak LG**	**H2 Peak LG**	**No H2 Peak SO**	**H2 Peak SO**
1	6.113	3.440	4.716	2.670	4.110	1.969	3.805	2.440
2	1.403	2.326	6.714	5.434	4.256	4.764	2.419	3.717
3	6.258	2.538	4.379	4.581	3.917	4.888	3.245	3.780
4	1.775	10.588	5.545	4.447	5.353	10.908	3.760	4.772
5	2.766	2.075	6.445	5.864	2.766	2.075	4.003	3.449
6	1.954	2.555	4.421	3.000	1.369	1.711	1.882	1.636
7	1.887	1.439	5.936	5.174	2.866	2.186	2.865	2.673
Mean ± 1 SD	3.165 ± 2.104	3.566 ± 3.154	5.451 ± 0.964	4.452 ± 1.210	3.519 ± 1.293	4.072 ± 3.302	3.141 ± 0.792	3.209 ± 1.036
95% Confidence Interval	1.606–4.724	1.229–5.902	4.736–6.165	3.556–5.349	2.561–4.477	1.626–6.518	2.554–3.727	2.442–3.977
T score	0.261		−3.713		0.600	0.195		
P value	0.803		0.009		0.570	0.852		
Effect Size	−0.15	0.913		−0.22	−0.074			

*All EMG values are in normalized units.

#### 1 Dimension Results

**Figure 3:**
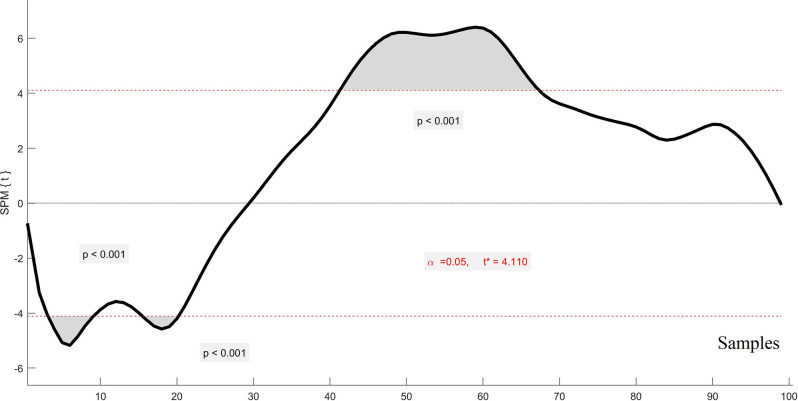
An exemplary statistical parametric mapping (SPM) waveform displaying significant effects of wearing the H2 on the COP. The shaded areas represent portions of the COP waveform where statistically significant differences occurred. The *p* < 0.05 value is represented by the dotted lines at 4 and -4 on the ordinate. Perturbation onset occurred at 20 on the abscissas.

Figure 3 shows an exemplary outcome of SPM testing for a participant whose COP was affected by wearing the H_2_.

#### SPM Outcomes

**Figure 4:**
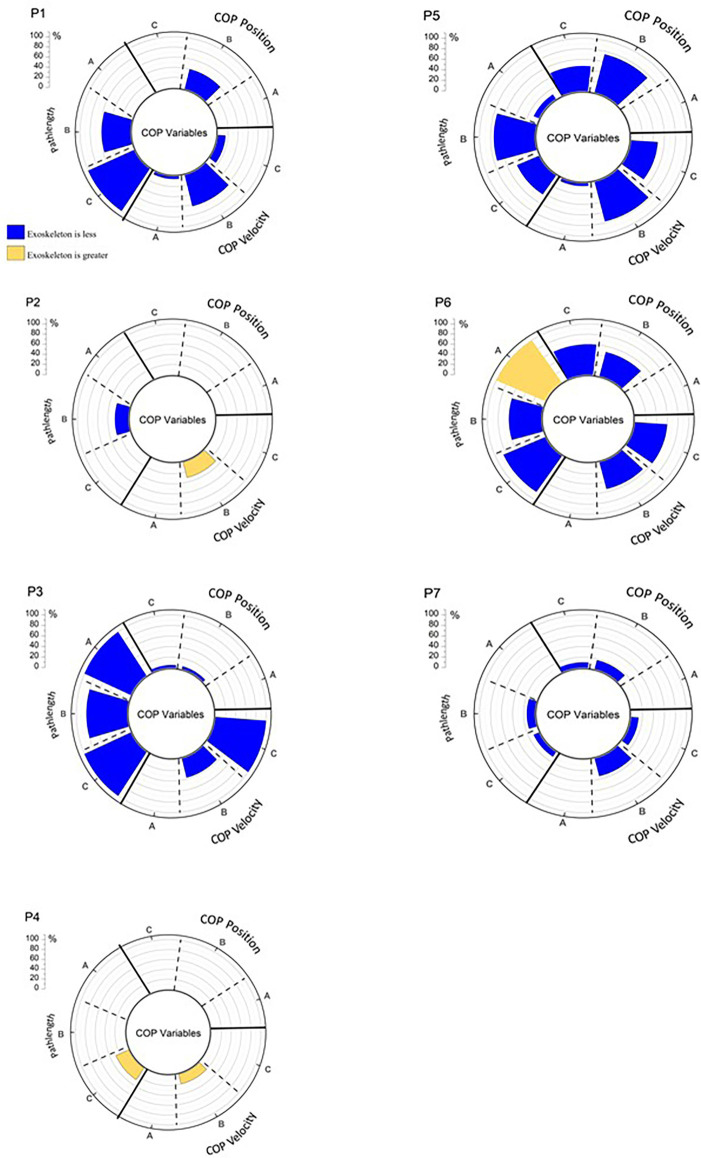
The percentage of significant SPM testing t-values for each analysis epoch for COP by participant. A = Baseline, B = Response, C = Recovery, P = participant number. Values in blue represent that the value obtained while wearing the H2 is less than when obtained while not wearing the H2. Values in gold, represent the opposite direction of change. If there were no significant difference between wearing and not wearing the H2 there is no data represented on the chart for a given variable and epoch.

**Figure 5:**
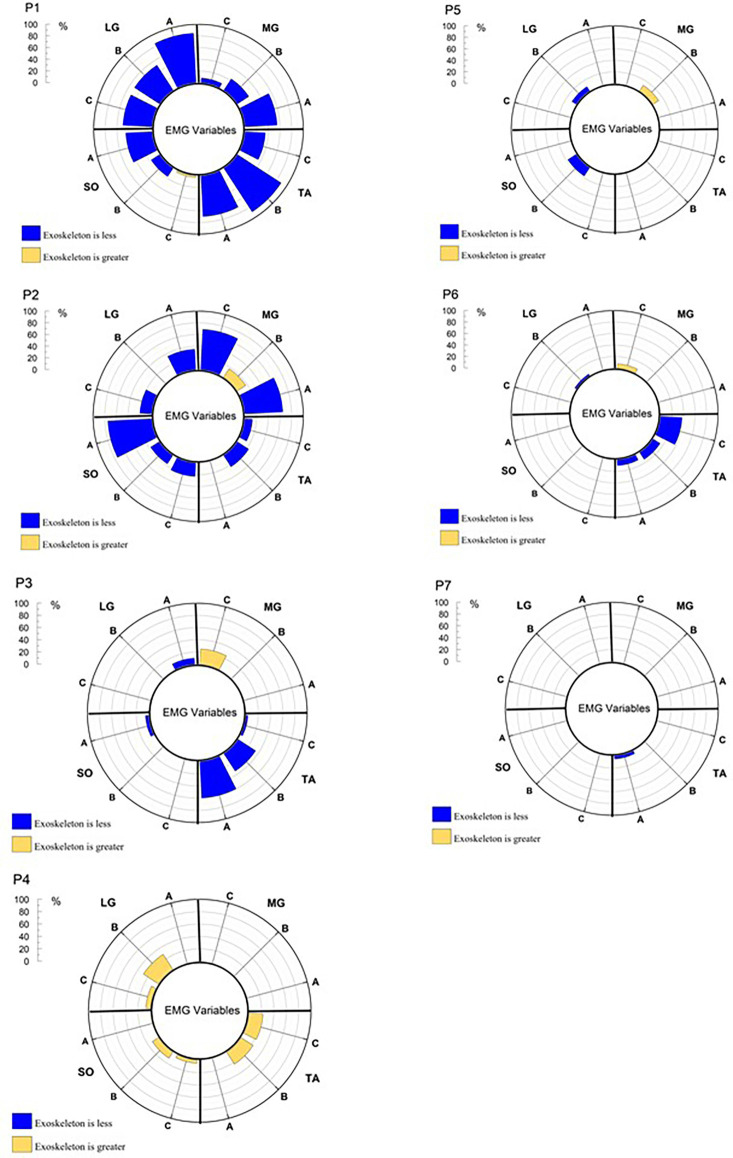
The percentage of significant SPM testing t-values for each analysis epoch for the EMG activation waveforms, by muscle and participant.

Figure 4 displays the percentage of COP variable samples in a particular analysis epoch that were significantly different during testing with and without wearing the H2. It is readily apparent that wearing the H2 impacts each of the participants in a unique manner. These individual analyses are an important feature provided by SPM relative to traditional 0D analyses. That being said, while SPM does provide samples that are statistically different between waveforms, knowledge of the direction of difference (i.e., did wearing the H2 result in greater or less magnitude of a given variable) is needed to more completely understand the impact of the H2. Therefore, the direction of change is also represented in Figure 4 by color. Figure 5 displays the results of SPM analyses, for each epoch, for the four monitored muscles. The color-coding representing the direction of difference is the same as in Figure 5.

Participant 1 showed relatively high percentages of significant differences across the COP (Figure 4) and muscle activation waveforms (Figure 5) in all three analysis epochs. Participants 5 and 6 displayed a number of significant differences between the COP waveform, but few in the EMG waveforms. Participant 7 shows a similar pattern of differences, but the percentage of samples that are significantly different is much fewer than those observed for Participants 5 and 6. In contrast, Participant 3 displays many significant changes in EMG waveforms across multiple epochs, but few changes in the COP variables. It should be further noted that some participants displayed reduced responses while wearing the H2 while others displayed greater responses. Similarly, the direction of change may differ between the COP and EMG variables. Figure 4 also illustrates that six of seven participants display differences in the Response and Recovery epochs for pathlength. Additionally, all participants display some significant differences in COP velocity between the H2 and No H2 conditions during the Response epoch. In summary, SPM analysis of the 1D waveforms effectively revealed that all participants *were* impacted by wearing the H2 exoskeleton, but—importantly—that each participant displayed different patterns of behavior and neuromuscular activation in response to the perturbations.

## Discussion

When exploring potential changes resulting from wearing an exoskeleton, there are several important factors to consider. Many exoskeletons, including the H2, add significant additional mass that must be adapted to and controlled for. The addition of mass to the human body can lead to compensatory changes in positioning (Singh and Koh, 2009) as well as alter inertial characteristics (Haddox et al., 2020). Many exoskeletons will increase the base of postural support (BOS) which, under typical circumstances, will tend to increase postural stability; however, this may not always be the case with all exoskeletons. An exoskeleton whose CoM exists only below its user’s natural CoM will inherently lower its user’s CoM. In that sense, any tethered, external mass added below an individual’s CoM will drive their CoM inferiorly. However, this translation of the CoM does not necessarily imply greater postural stability; more likely the user needs to adopt a new control strategy that accounts for the altered COM. Given the importance of the CoM motion and position to both standing stability and locomotion (Shimba, 1984; Winter and Eng, 1995; Rajachandrakumar et al., 2018), this is an important avenue for future research, as well as an important consideration for exoskeleton manufacturers.

The H2 also includes foot pads as contact surfaces with the ground that could interfere with or modulate cutaneous and proprioceptive inputs normally used to control balance. Finally, depending upon the amount of structural support offered by an exoskeleton, muscle activation patterns, and their associated torques may need to be modified, *via* motor learning (Zhu et al., 2021), in order to maintain stability. How these factors uniquely interact with the user will determine the responses observed during both quiet stance and postural perturbations.

### Effect of H2 During Static Balance Testing

The COP RMS measures of the 2-min static baseline condition indicate there was not a systematic effect of wearing the H2 during a quiet stance. Some participants exhibited greater sway, while others swayed less. This group-level finding is consistent with the findings of Ringhof et al. (2019), which identified no influence of their exoskeleton on bipedal quiet stance. It does, however, contrast with those of Schiffman et al. (2008), who found a significant decrease in COP sway. As in the current study, the participants in both Ringhof et al. (2019) and Schiffman et al. (2008) were healthy, young adults. It is likely that differences in both exoskeleton design and data collection procedures (e.g., assessment time, arm positioning, perturbation characteristics) could affect the results. In this study, all but one participant demonstrated an increase in peak COP velocity, which may suggest that the H2 could subtly impact the subtle motor control necessary to minimize sway during static balance.

### Effect of H2 in Response to Perturbations

The first observation to note is that in this study, none of the participants felt the need to take a step to maintain their balance, with or without the H2. This is consistent with the findings of Fasola et al. (2019), which also provided perturbations to young, healthy participants and observed no falls while wearing an exoskeleton. The current findings indicate the H2 mechanical structure and physical human-robot interface *via* the cuffs appears to provide enough stability to the user to allow for successful balance responses to external perturbations, at least with healthy participants.

The results from the statistical tests of the 0D variables indicate there is a significant behavioral effect of wearing the H2 during the responses to posterior perturbations. The combination of reduced peak COP position, velocity, and pathlength strongly suggests that the wearing of the H2 restricts the magnitude and velocity of sway associated with perturbation responses, relative to not wearing the exoskeleton. This reduced sway is not a function of the increased weight provided by the H2, as the Balance Master adjusts each perturbation for the increased weight of the device, such that the perturbation characteristics remain the same with or without the H2. The reduction in sway appears to be associated with the restriction of kinematic degrees of freedom that are available to participants during the perturbation. The inability to move the hip, knee, and ankle joints through their natural range of motion modulates neuromuscular activation patterns thereby influencing the coordination of postural response, as reflected in the altered COP.

Of particular interest is the fact that the participants utilized different neuromuscular activation strategies in response to the same perturbations. These strategies effectively worked to achieve the same goal—the maintenance of standing balance—but did so by utilizing a variety of different kinematic and electromyographic combinations. These findings would have been missed, had SPM testing not been performed in combination with the traditional 0D analysis. Figure 4 reveals that all participants did display significant changes in at least some COP parameters. Figure 5 reveals that some participants displayed many differences in neuromuscular activation while in the exoskeleton, while others showed very few differences. These results reflect that responses to perturbations while wearing an exoskeleton are highly individualized and that a variety of analytical measures are valuable in identifying unique response patterns.

## Limitations

The current study features a relatively low number of participants, limiting the degree of generalizability of these results. However, despite low participant numbers, our analysis demonstrated group statistical differences in several behavioral variables (e.g., COP) as a result of wearing the H2. SPM analysis also demonstrated robust differences between wearing and not wearing the H2, while also identifying personalized behavioral and neuromuscular response patterns. As only one model of the exoskeleton was used in this study, these results should be cautiously applied when considering other exoskeleton models. Results should be carefully applied to any other model of exoskeleton. The H2 does, however, share many similar features to other exoskeletons currently on the market. In particular, and by design, exoskeletons limit the available degrees of freedom and joint ranges of motion, which are likely mechanisms for the differences we identified between the two exoskeleton conditions. Moreover, the physical interface between the user and the robot, which includes cuffs to secure the exoskeleton and which may result in variant levels of compliance due to soft tissue (Bayón et al., 2022), is also likely to affect the responses to the postural perturbations.

## Conclusions

This investigation has revealed that wearing the H_2_ exoskeleton does impact responses to posterior support surface translations, as reflected in decreased magnitude and velocity COP responses. This appears to be primarily the result of restricted lower limb joint motion and the compliance of the physical robot-user interface, thereby modulating the coordination patterns available in response to the perturbation. Despite this, all participants were able to develop effective responses that enabled them to maintain their stability without falling or even requiring a step. These coordination patterns varied greatly by the participant. Likewise, there was significant intraindividual variability in the neuromuscular responses to perturbation. This again points to unique, individualized approaches to the maintenance of stability. We believe our results provide compelling evidence that robotic exoskeleton users will interact with the same exoskeleton device in a unique manner. Developers should seek to maximize the number of individualized features on their exoskeleton systems in order to best tailor and adapt their devices to the morphology and responses of the end-user.

## Data Availability Statement

The raw data supporting the conclusions of this article will be made available by the authors, without undue reservation.

## Ethics Statement

The studies involving human participants were reviewed and approved and the experimental protocol was approved by the Institutional Review Board (IRB) at the University of Houston, in accordance with the Declaration of Helsinki. The patients/participants provided their written informed consent to participate in this study.

## Author Contributions

CL was responsible for study conceptualization, data collection and analysis, and writing the text of the manuscript. CM was responsible for study conceptualization, data collection, Matlab script development, data analysis, and manuscript preparation. AR was responsible for Matlab script development and data analysis. IJ was responsible for data collection and analysis. GF was responsible for manuscript review. JC-V was responsible for study conceptualization and manuscript preparation. All authors contributed to the article and approved the submitted version.

## Conflict of Interest

The authors declare that this research was supported in part by industry membership fees through the IUCRC BRAIN—an industry-university-government partnership to accelerate the development of neurotechnologies. The authors declare that they have no competing interests.

## Publisher’s Note

All claims expressed in this article are solely those of the authors and do not necessarily represent those of their affiliated organizations, or those of the publisher, the editors and the reviewers. Any product that may be evaluated in this article, or claim that may be made by its manufacturer, is not guaranteed or endorsed by the publisher.
